# Phytochemical Profile and Antioxidant Activity of *Lavandula angustifolia* and *Lavandula* x *intermedia* Cultivars Extracted with Different Methods

**DOI:** 10.3390/antiox11040711

**Published:** 2022-04-05

**Authors:** Natalia Dobros, Katarzyna Zawada, Katarzyna Paradowska

**Affiliations:** Department of Physical Chemistry, Chair of Physical Pharmacy and Bioanalysis, Faculty of Pharmacy, Medical University of Warsaw, Banacha Str. 1, 02-097 Warsaw, Poland; natalia.dobros@wum.edu.pl (N.D.); katarzyna.zawada@wum.edu.pl (K.Z.)

**Keywords:** lavender, lavandin, aqueous-ethanolic and aqueous extracts, bioactive compounds, qualitative and quantitative HPLC analysis, free radicals

## Abstract

Lavender is a valuable perennial plant from the Lamiaceae family. It is grown mainly for its essential oil, but it also contains polar bioactive compounds such as polyphenols and coumarins. Their level depends on the species, cultivars, geographical origin, climatic conditions, harvest time and extraction method. The authors investigated the effect of several extraction procedures (maceration, decoction and ultrasound-assisted extraction) applied to three cultivars of *Lavandula angustifolia* (Betty’s Blue, Elizabeth, Hidcote) and two cultivars of *Lavandula* x *intermedia* (Grosso, Gros Bleu) on the yield of the polyphenolic compounds and antioxidant activity. HPLC analysis showed the presence of rosmarinic acid (2.52–10.82 mg/g), ferulic acid glucoside (2.94–8.67 mg/g), caffeic acid (1.70–3.10 mg/g), morin (1.02–13.63 mg/g), coumarin (1.01–5.97 mg/g) and herniarin (1.05–8.02 mg/g). The content of phenolic acids and flavonoids was higher in lavender, while the content of coumarins was higher in lavandin in all types of extracts. The antioxidant activity was determined by DPPH-EPR assay for antiradical properties (104.58–206.77 μmol Trolox/g) and FRAP assay for reducing properties (79.21–203.06 μmol Trolox/g). The obtained results showed that the cultivar is the dominant factor differentiating the samples. Still, the extraction method plays an important role in the final bioactive substances content and antioxidant properties of obtained extracts.

## 1. Introduction

Lavender is a highly valuable perennial plant belonging to the Lamiaceae family. The genus *Lavandula* consists of 39 species. Lavender (*Lavandula angustifolia* Mill.), lavandin (*Lavandula intermedia* Emeric ex Loisel., which is a cross between *L. angustifolia* and *L. latifolia*), spike lavender (*Lavandula latifolia* Medik.), *Lavandula stoechas* and *Lavandula dentata* are among the most widely cultivated species of this genus [1,2,3]. In traditional medicine, lavender is used to treat numerous diseases owing to its sedative [4], antidepressant [5], spasmolytic, anticholinesterases [6], anti-inflammatory [7,8,9,10], antibacterial [3], antifungal and antioxidant properties [11,12,13,14,15].

Lavender is cultivated mainly for its essential oil, but it also contains many other compounds such as polyphenols, coumarins, triterpenes, sterols and tannins [16]. Polyphenols have antioxidant activity owing to their ability to scavenge free radicals, chelate metal ions (e.g., Fe^2+^, Cu^2+^) and inhibit the activity of pro-oxidative enzymes [17]. Polyphenols are secondary plant metabolites that are classified into several groups, such as phenolic acids, flavonoids, lignans and stilbenes [18]. Phenolic compounds of lavender flowers include hydroxybenzoic acids (p-hydroxybenzoic acid, protocatechuic acid, vanillic acid, gentisic acid, gallic acid), hydroxycinnamic acids (rosmarinic acid, caffeic acid, p-coumaric acid, ferulic acid, chlorogenic acid, sinapic acid, cinnamic acid, 4-*O*-caffeoylquinic, 5-*O*-caffeoylquinic) and flavonoids (apigenin and luteolin glycosides, catechin, naringenin, vanillin) [3,5,6,14,19,20]. New phenolic compounds such as lavandunat, lavandufurandiol, lavandufluoren, lavandupyrones A and B, lavandudiphenyls A and B were isolated by Yadikar et al. [21]. The level of each of these constituents varies in different species and depends on the genotype, geographical origin, climatic conditions, growing conditions, harvest time and extraction method [12].

For the extraction of active compounds from plant materials, conventional and modern techniques are used. The conventional methods, including solid-liquid extraction, maceration [6,22], decoction [23], Soxhlet extraction or hydrodistillation, are applied generally for galenical preparations. They require large quantities of solvents, extended extraction times and may contribute to the degradation of thermolabile compounds. On the other hand, modern methods such as ultrasound-assisted extraction (UAE), microwave-assisted extraction (MAE), pressurised liquid extraction (PLE), accelerated solvent extraction (ASE) and supercritical fluid extraction (SFE) are more environmentally friendly and thus have been named “green techniques” [24,25]. The yield of active compounds depends on the extraction method, the fragmentation of raw material and the properties of the extraction solvent [26]. According to Costa et al. [14], water and the ethanol-water mixture are the most suitable solvents for the extraction of phenolic compounds. Other factors such as extraction steps, extraction time, temperature and the solvent to sample ratio may additionally affect the extractability of polyphenolics [24,27].

This study aimed to determine the impact of the extraction methods (maceration, decoction and ultrasound-assisted extraction) and cultivars of lavender (*Lavandula angustifolia*) and lavandin (*Lavandula* x *intermedia*) on the content of phenolic acids, flavonoids, coumarins and antioxidant activity of the extracts. Ferric reducing antioxidant power assay (FRAP) and free radical scavenging assay (DPPH-EPR-electron paramagnetic resonance test) were used to determine the antioxidant activity of plant extracts. The relationships between total polyphenols, total flavonoids and antioxidant activity were also analysed using Pearson’s correlation coefficient and principal component analysis (PCA).

## 2. Materials and Methods

### 2.1. Materials (Chemicals)

Folin-Ciocalteu reagent, gallic acid (3,4,5-trihydroxybenzoic acid), ethanol 96%, methanol 99.8%, sodium nitrite, aluminium chloride, sodium carbonate, sodium hydroxide, iron (III) chloride were purchased from Avantor Performance Materials Poland S.A (Gliwice, Poland), whereas DPPH (2,2′-diphenyl-1-picrylhydrazyl), TPTZ (2,4,6-tripyridyl-s-triazine), (+)-catechin, chlorogenic acid, caffeic acid, rosmarinic acid, morin and herniarin (7-methoxycoumarin) were from Sigma-Aldrich (Steinheim, Germany). Coumarin was obtained from Acrōs Organics (Fisher Scientific), while trans-ferulic acid, ellagic acid, vanillin and isoquercitrin (quercetin 3-glucoside) were from PhytoLab GmbH & Co. KG (Vestenbergsgreuth, Germany). Trolox (6-hydroxy-2,5,7,8-tetramethylchroman-2-carboxylic acid), formic acid and HPLC-grade acetonitrile were acquired from Merck (Darmstadt, Germany). Purified water (“MilliQ water”) was obtained from a MilliQ System (System Direct-Q^®^ 3UV Ultrapure (type 1) water) from Merck (Darmstadt, Germany). All chemicals and reagents were of analytical reagent grade.

### 2.2. Plant Material

The inflorescences of different cultivars of lavender (*Lavandula angustifolia* Mill.) and lavandin (*Lavandula* x *intermedia* Emeric ex Loisel.) were harvested from the plantation “Przystanek Lawenda” (which possesses the status of National Collection of the genus *Lavandula*) located in Borowiczki-Pieńki near Płock (Mazovia Province) in the central part of Poland. The voucher specimens were deposited in the Herbarium Universitatis Varsoviensis (Warsaw, Poland) with voucher numbers WA0000111924 (Hidcote), WA0000111925 (Elizabeth), WA0000111926 (Betty’s Blue), WA0000111927 (Gros Bleu), WA0000111928 (Grosso). Plant material was collected during the flowering season in June 2019. Lavender inflorescences were subjected to a freeze-drying process (Alpha 1-2 LDplus CHRIST, Donserv, Warsaw, Poland) at −25 °C, 0.63 mbar, for 50 h, and then ground in an electric milling machine. The powdered samples were stored in a dry, cool and dark place until further analysis.

### 2.3. Extraction Procedure

#### 2.3.1. Preparation of Macerates

Macerates were prepared according to the procedure described in the Polish Pharmacopoeia [28]. One gram of plant material was soaked without mixing in 20 mL of 50% ethanol for 30 min at room temperature. After maceration, the extracts were centrifuged at 3000 rpm for 10 min and filtered off. All samples were prepared in triplicate.

#### 2.3.2. Preparation of Decoctions

Decoctions were prepared according to the procedure described in the Polish Pharmacopoeia [28]. Ten millilitres of MilliQ water was added to 1 g of plant material and boiled for 30 min. Water was systematically added during cooking, and finally, decoctions were filled to a 10 mL volume. Next, the extracts were centrifuged at 3000 rpm for 10 min and filtered off. All samples were prepared in triplicate.

#### 2.3.3. Preparation of Ultrasound-Assisted Extracts (UAE)

Ultrasound-assisted extraction was performed according to the modified method described by Arceusz and Wesolowski [29]. One gram of plant material was mixed with 20 mL of 50% ethanol and sonicated for 30 min, in the first stage, with 10 mL and for 15 min in the second and third stages. During the whole sonication process, the temperature did not exceed 36 °C. The combined extracts were centrifuged at 3000 rpm for 10 min and filtered off. All samples were prepared in triplicate.

One part of the obtained supernatants (4 mL) was placed in Eppendorf tubes and stored at −32 °C until total polyphenol, total flavonoid and antioxidant analyses. The second part of the supernatants was evaporated to dryness using a rotary evaporator (Heidolph Instruments, Schwabach, Germany). Then, the dry samples were dissolved in 10 mL of MilliQ water and freeze-dried at −25 °C, 0.63 mbar for 50 h. Thus, obtained samples were used for HPLC analysis.

### 2.4. Total Phenolic Content (TPC)

The total phenolic content (TPC) was determined using the modified Folin-Ciocalteu colourimetric method, as previously described by Waterhouse [30]. Briefly, 15 μL of the extract solution or gallic acid solution was diluted with 1185 μL of MilliQ water and subsequently, 75 μL of Folin-Ciocalteu reagent was added and mixed on a vortex. After 2 min, 225 μL of 20% sodium carbonate was added, and the reaction mixture was thermostatted for 20 min at 40 °C. The absorbance was measured against the blank at 765 nm using an Evolution 60S UV-Visible spectrophotometer (Thermo Scientific, Waltham, MA, USA). All samples were analysed in triplicate. The results were expressed as milligrams of gallic acid equivalents (GAE) per gram of dry matter of plant material (mg GAE/g d.w.) based on the calibration curve: y = 0.0011x + 0.0016, R^2^ = 0.999 (mg/L).

### 2.5. Total Flavonoid Content (TFC)

The total flavonoid content (TFC) was determined using the modified colourimetric method described by Jindřiška and Neugebauerová [31]. The 1.4 mL of MilliQ water, 100 μL of an extract solution or a catechin solution, 60 μL of 5% sodium nitrite and 60 μL of 10% aluminium chloride were mixed. After 5 min of incubation at 25 °C, 0.4 mL of 1 M sodium hydroxide solution was added. The solution was mixed thoroughly, and the absorbance was measured against a blank solution at 510 nm using an Evolution 60S UV-Visible spectrophotometer (Thermo Scientific, Waltham, MA, USA). All samples were analysed in triplicate. The results were expressed as milligrams of catechin equivalents (CA) per gram of dry matter of plant material (mg CA/g d.w.) based on the calibration curve: y = 0.0016x + 0.0053, R^2^ = 0.998 (mg/L).

### 2.6. Phytochemical Profile Analysis

#### 2.6.1. HPLC-DAD-UV/VIS Analysis

##### Preparation of Standard Curves

The stock solutions of chlorogenic acid, caffeic acid, ferulic acid, ellagic acid, rosmarinic acid, vanillin, isoquercitrin, morin, coumarin and herniarin, selected as the most common in plant material, were prepared by dissolving 1 mg of standard in 1 mL of methanol, then mixed together and filtered through a 0.45 μm syringe filter (Bionovo, Legnica, Poland). The concentrations of the most abundant compounds (caffeic acid, ferulic acid glucoside, rosmarinic acid, morin, coumarin and herniarin) were determined using an appropriate calibration curve. All measurements were performed in triplicate.

The linearity, detection limit and quantification limit were calculated from the curve to validate the method. The linearity was evaluated by the value of the coefficient of determination (R^2^) of the calibration curve acquired for each standard. The limit of detection (LOD) and the limit of quantification (LOQ) were determined using the equations: LOD = 3.3 × σ/S and LOQ = 10 × σ/S, respectively (where σ is the standard deviation of the y-intercept and S is the slope of the calibration curve) (Table S1). The precision of the method was determined by injecting three concentrations of standards: 40, 80 and 200 μg/mL for rosmarinic acid and herniarin, three times on the same day and the next day. The intraday and interday precision were assessed by calculating the relative standard deviation (RSD) (Table S2). The coefficient of determination (R^2^) for each compound was higher than 0.997, which indicates a high correlation of the data in the analysed concentration range. The LODs were from 0.008 to 0.021 mg/mL, while the LOQs were from 0.024 to 0.065 mg/mL (Table S1). The RSD values for the intraday and interday precision were below 1%, which indicated a good precision of the method (Table S2).

##### Extract Analysis

Qualitative and quantitative analyses of the lavender and lavandin extracts were performed using HPLC-DAD-UV/VIS. Separation of phenolic compounds and coumarins was achieved using the Hitachi Chromaster system (Tokyo, Japan) with a Purospher STAR RP-18e column (5 μm particle size, 250 mm × 4.6 mm, Merck) at 30 °C. The autosampler temperature was set at 20°C, and the sample injection volume was 20 μL. The mobile phase consisted of 0.1% (*v*/*v*) formic acid in water (A) and 0.1% (*v*/*v*) formic acid in acetonitrile (B) at a flow rate of 1 mL/min. The gradient conditions were as follows: 10–20% B (0–35 min), 20–35% B (35–60 min), 35–10% B (60–60.1 min), 10% B (60.1–70 min). The freeze-dried extracts (40 mg) were dissolved in 2 mL of 50% ethanol (ultrasound-assisted extracts and macerates) or water (decoctions). The chromatograms were recorded at 250, 280 and 330 nm. The peaks were identified by comparing their retention times and UV-Vis spectra with standards.

#### 2.6.2. MS Analysis

Mass spectrometry analyses were carried out using ultra-performance liquid chromatograph ACQUITY UPLC I-Class (Waters) coupled with Synapt G2-S mass spectrometer (Waters) equipped with the electrospray ion source and quadrupole-time-of-flight mass analyser. The resolving power of the TOF analyser was 40000 FWHM. The recorded data were processed using the MassLynx V4.1 software package (Waters). The chromatographic separations were performed on a Waters Acquity UPLC BEH C18 column (1.7 μm particle size, 2.1 mm × 100 mm). The mobile phase consisted of 0.1% (*v*/*v*) formic acid in water (A) and methanol (B) at a flow rate of 0.30 mL/min. The gradient conditions were as follows: 5% B (0–3 min), 5–100% B (3–30 min), 100-5% B (30–33 min), 5% B (33–35 min). The sample injection volume was 2 μL, and the wavelength of the UV detector was set at 254 nm and 280 nm. The mass spectrometry measurement was achieved both in the positive and negative ion modes. The measurements were performed with capillary voltage set to 2.94 kV in positive ion mode and 3.05 kV in negative ion mode. The desolvation gas flow was 799 L/h and temperature 450 °C, and 774 L/h and temperature 350 °C, in positive ion mode and negative ion mode, respectively. The sampling cone voltage and source offset were set to 20 V in positive ion mode, while in negative ion mode, they were set to 32 V. The source temperature was 120 °C in both cases. The Leucine–Enkephaline solution was used as the Lock-Spray reference material. Commercial libraries such as MassBank (High-Quality Mass Spectral Database) and NIST Chemistry WebBook were used to identify compounds.

### 2.7. DPPH Scavenging (Electron Paramagnetic Resonance Test)

The free radical scavenging activity of the extracts was measured in vitro by the 2,2′-diphenyl-1-picrylhydrazyl (DPPH) assay described by Sanna et al. [32]. The stock solution was prepared by dissolving 12.5 mg DPPH in 25 mL methanol and stored at 4 °C until required but no longer than 12 h. In the experiment, 20 μL of the sample or blank was mixed with 180 μL of DPPH solution and kept for 30 min in the dark. EPR spectra were recorded, and their intensity was assumed as the double integral of the spectra. All samples were analysed in triplicate. The results were expressed as micromoles of Trolox equivalents per gram of dry matter of plant material (μM Trolox/g d.w.). EPR measurements were performed using a MiniScope MS200 spectrometer (Magnettech GmbH, Berlin, Germany) with the following set of parameters: central field 330 mT, sweep range 9.9 mT, sweep time 20 s, microwave power 6 mW, modulation amplitude 0.15 mT.

### 2.8. The Ferric Reducing Antioxidant Power (FRAP Assay)

The ferric reducing antioxidant power (FRAP) assay was performed according to the modified procedure described by Benzie and Strain [33]. Briefly, 50 μL of the sample or Trolox solution was mixed with 1000 μL of working FRAP reagent. The reaction mixture was thermostatted for 4 min at 37 °C. The absorbance was measured against the blank solution at 593 nm using an Evolution 60S UV-Visible spectrophotometer (Thermo Scientific, Waltham, MA, USA). The working FRAP reagent was prepared by mixing 20 mM iron (III) chloride and 10 mM TPTZ solutions with 300 mM acetate buffer (pH 3.6) in a proportion of 1:1:10, respectively. All samples were analysed in triplicate. The results were expressed as micromoles of Trolox equivalents per gram of dry matter of plant material (μM Trolox/g d.w.) based on the calibration curve: y = 0.0021x − 0.0108, R^2^ = 0.998 (μM/L).

### 2.9. Statistical Analysis

The results were expressed as mean ± SD (n = 3). One-way analysis of variance (ANOVA) coupled with the Tukey post-hoc test was used to compare the data and to identify means with significant differences (*p* ˂ 0.05). Pearson’s correlation coefficient (r) was used to assess the relationship between antioxidant activity and the phenolic compound content at a 5% probability level. All data were subjected to PCA analysis as well. All statistical analyses were carried out with the TIBCO Statistica 13.3 (StatSoft Poland).

## 3. Results and Discussion

**Figure 1:**
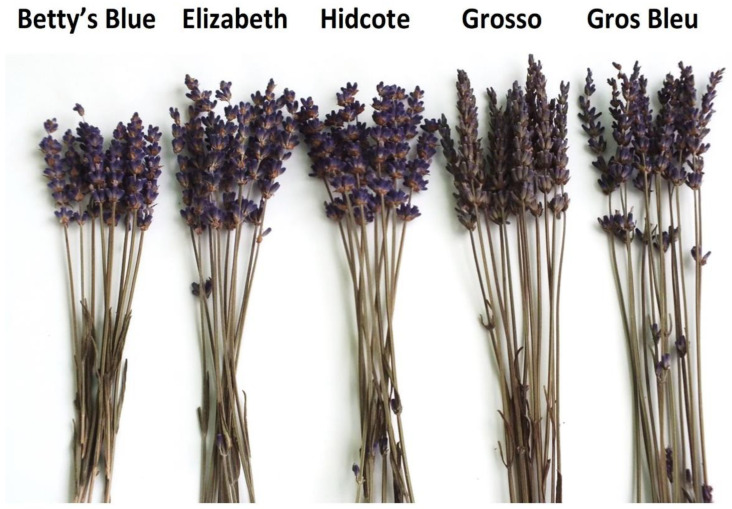
The inflorescences of different cultivars of lavender (*Lavandula angustifolia* Mill.) and lavandin (*Lavandula* x *intermedia* Emeric ex Loisel.).

The typical appearances of the inflorescences of studied cultivars of lavender (*Lavandula angustifolia* Mill.) and lavandin (*Lavandula* x *intermedia* Emeric ex Loisel.) are shown in Figure 1.

### 3.1. Total Phenolic and Flavonoid Content

**Figure 2:**
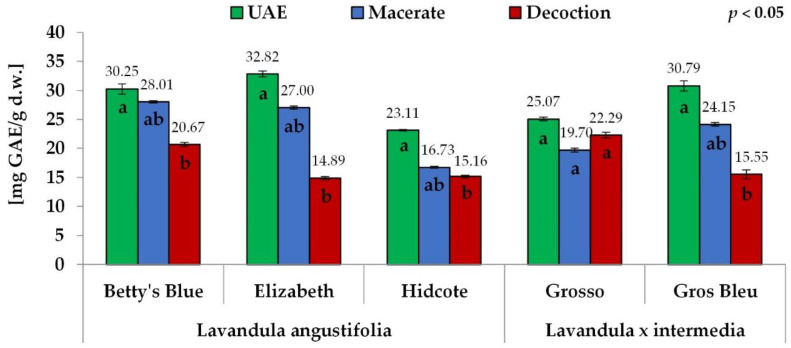
The total phenolic content for different extraction methods. The values with different letters within the column designate statistically significant differences, *p* < 0.05 by ANOVA. Statistical analysis was performed separately for each cultivar.

**Figure 3:**
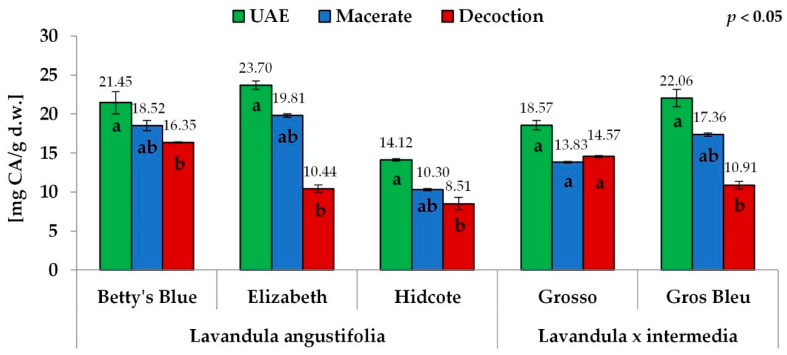
The total flavonoid content for different extraction methods. The values with different letters within column designate statistically significant differences, *p* < 0.05 by ANOVA. Statistical analysis was performed separately for each cultivar.

In this study, the total phenolic content (TPC) varied from 14.88 to 32.82 mg GAE/g d.w. for *Lavandula angustifolia* extracts and from 15.55 to 30.79 mg GAE/g d.w. for *Lavandula* x *intermedia* (Figure 2). The total flavonoid content (TFC), a subgroup of polyphenols, ranged between 8.51 and 23.70 mg CA/g d.w. for *Lavandula angustifolia* extracts and between 10.91 and 22.06 mg CA/g d.w. for *Lavandula* x *intermedia* (Figure 3). Statistically significant differences were found between the extraction methods, especially between an ultrasound-assisted method and decoction, for the total phenolic content and total flavonoid content for both species. An ultrasound-assisted method (UAE) gave the highest values of the total phenolic content (23.11–32.82 and 25.07–30.79 mg GAE/g d.w., for *Lavandula angustifolia* and *Lavandula* x *intermedia* extracts, respectively), followed by maceration (16.73–28.01 and 19.69–24.15 mg GAE/g d.w.) and decoction (14.88–20.67 and 15.55–22.28 mg GAE/g d.w.) (Figure 2).

Similar to the total phenolic content, the aqueous-ethanolic extracts of both species showed a higher total flavonoid content than the aqueous extracts. The highest total flavonoid content was observed for the extracts obtained by the ultrasound-assisted method (14.12–23.70 and 18.57–22.06 mg CA/g d.w. for *Lavandula angustifolia* and *Lavandula* x *intermedia* extracts, respectively), followed by maceration (10.30–19.81 and 13.83–17.36 mg CA/g d.w.) and then the decoction (8.51–16.35 and 10.91–14.57 mg CA/g d.w.) (Figure 3). The values of total phenolic and total flavonoid contents obtained for *Lavandula* x *intermedia* for the Grosso cultivar, with higher results for decoction (22.28 mg GAE/g d.w. and 14.57 mg CA/g d.w.) than macerate (19.69 mg GAE/g d.w. and 13.83 mg CA/g d.w.), were the exception (Figure 2, Figure 3).

Our results are in agreement with those reported by Tušek et al. [34], where the total phenolic content of *Lavandula* x *intermedia* was 17.83 mg GAE/g d.w. and 26.21 mg GAE/g d.w. when the extraction was performed with water at 40 °C and 60 °C, respectively. Likewise, Jiménez-Zamora et al. [11] obtained a result equal to 322 mg GAE/L, which corresponds to 24.15 mg GAE/g d.w., for the lavender infusion. Yet, these results are higher than those reported by Kontogiorgis [23]. In their study, the total phenolic content for decoction prepared from *Lavandula angustifolia* was 130.59 mg GAE/L, which corresponds to 3.92 mg GAE/g d.w. This could be due to a shorter extraction time of 5 min as compared with the 30 min extraction time in this work. Moreover, research on methanolic extracts from 30 different populations of *Lavandula* x *intermedia* indicated the presence of phenolic compounds in the range of 0.31–1.05 mg GAE/g d.w., with the flavonoid content of 0.28–0.72 mg QE/g d.w. [12]. On the other hand, in the study of Spiridon et al. [35], the extraction of lavender with methanol afforded a higher total phenolic content equal to 50.6 mg GAE/g as well as a higher total flavonoid content equal to 27.6 mg rutin/g. In addition, our results were lower than those obtained by Hawrył et al. [13], where the total phenolic content of methanolic extracts was 1.46 mg GAE/mL (which could be recalculated to 72.87 mg GAE/g d.w. for plant material) for *Lavandula angustifolia* Atropurpurea and 1.98 mg GAE/mL (98.88 mg GAE/g d.w.) for *Lavandula angustifolia* Rosea. The above values are much higher than those reported in our study, probably because other extraction procedures and different varieties of lavender were used.

In general, the highest values of the total phenolic content (TPC) and total flavonoid content (TFC) were obtained for the Betty’s Blue and Elizabeth cultivar extracts, whereas the lowest ones were obtained for the Hidcote cultivar extracts. The order of other cultivars depended on the extraction method. In the case of extracts obtained by the ultrasound-assisted method (UAE), there were no significant differences between the Betty’s Blue, Elizabeth and Gros Bleu cultivars (TPC: 30.25; 32.82; 30.79 mg GAE/g d.w. and TFC: 21.45; 23.70; 22.06 mg CA/g d.w.). Nevertheless, the above cultivars had significantly higher total phenolic and total flavonoid contents than the Grosso and Hidcote cultivars (TPC: 25.07; 23.11 mg GAE/g d.w. and TFC: 18.57; 14.12 mg CA/g d.w., respectively) (Figure 2, Figure 3).

As concerns macerates, the highest total phenolic and total flavonoid contents were obtained again for the Betty’s Blue and Elizabeth cultivars (TPC: 28.01 and 27.00 mg GAE/g d.w., TFC: 18.52 and 19.81 mg CA/g d.w.). The order of the remaining samples observed for the total phenolic and flavonoid contents was the same: Gros Bleu, Grosso and Hidcote (TPC: 24.15; 19.70 and 16.73 mg GAE/g d.w. whereas for the TFC: 17.36; 13.83 and 10.30 mg CA/g d.w.). Among decoctions, the Grosso cultivar had the highest phenolic content (22.29 mg GAE/g d.w.). The Betty’s Blue cultivar (20.67 mg GAE/g d.w.) showed slightly lower values than the Grosso cultivar, but higher than those obtained for the Gros Bleu, Hidcote and Elizabeth cultivars (15.55; 15.16; 14.89 mg GAE/g d.w.). The highest total flavonoid content was obtained for Betty’s Blue cultivar (16.35 mg CA/g d.w.). In other cultivars, the observed total flavonoid contents were significantly lower in the order: Grosso, Gros Bleu, Elizabeth, Hidcote (14.57; 10.91; 10.44; 8.51 mg CA/g d.w.).

### 3.2. Determination of Phenolic Compounds and Coumarins by HPLC

**Figure 4:**
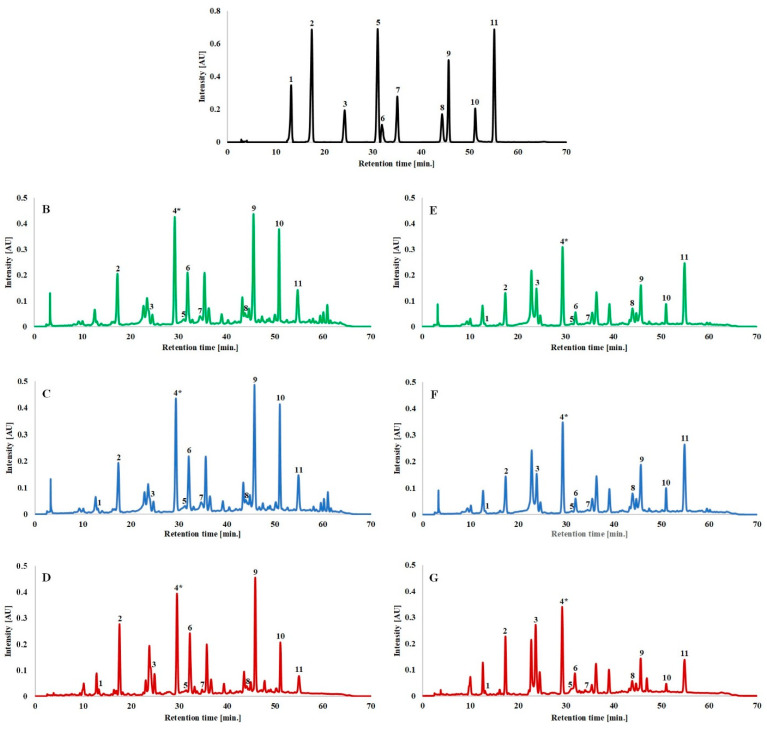
HPLC chromatograms of the standard mixture (**A**), lavender ((**B**–**D**) for Betty’s Blue cultivar) and lavandin ((**E**–**G**) for Gros Bleu cultivar) for UAE (green), macerate (blue) and decoction (red) at 330 nm. Numbers of compounds: 1—chlorogenic acid, 2—caffeic acid, 3—vanillin, 4 *—ferulic acid glucoside, 5—ferulic acid, 6—ellagic acid, 7—isoquercitrin, 8—coumarin, 9—rosmarinic acid, 10—morin, 11—herniarin. * Compound number 4 was identified using UPLC-MS analysis.

The representative HPLC chromatograms at 330 nm for lavender (Betty’s Blue) and lavandin (Gros Bleu) extracts (UAE), macerates and decoctions are presented in Figure 4. The main compounds (Figure S1) in all types of extracts from all cultivars of *Lavandula angustifolia* and *Lavandula* x *intermedia* were rosmarinic acid (no. 9), ferulic acid glucoside (no. 4), morin (no. 10), caffeic acid (no. 2) and herniarin (no. 11). Our results were similar to those obtained for *Lavandula angustifolia*, *Lavandula* x *intermedia* Budrovka [36], *Lavandula stoechas* [37,38], *Lavandula dentata* [37] and *Lavandula pedunculata* [39], where rosmarinic acid was the predominant compound. Ellagic acid (no. 6), coumarin (no. 8), isoquercitrin (no. 7) and vanillin (no. 3) were also identified, but in much smaller amounts. In turn, chlorogenic acid (no. 1) and ferulic acid (no. 5) were detected in trace amounts. On the other hand, Hawrył et al. [13] reported the presence of ferulic acid only in *Lavandula viridis* methanolic extract, while Adaszyńska-Skwirzyńska and Dzięcioł [40] only in *Lavandula angustifolia* leafy stalks.

The peaks were identified by comparing their retention times and UV spectra with standards and by the presence of a characteristic fragmentation pattern (Table S3, Figure S2). Only the identification of compound four was based solely on a fragmentation pattern analysis of UPLC-MS results since no standard was available. It was identified as ferulic acid glucoside based on fragment ions released at *m/z* 193.05, 149.06 and 134.04 (Figure S3). Previously, this compound has been found in *Lavandula angustifolia* aqueous-ethanolic extract [35], *Lavandula dentata* hydromethanolic extract [37] and *Lavandula* x *intermedia* waste [41] obtained after the steam distillation of essential oils.

**Figure 5:**
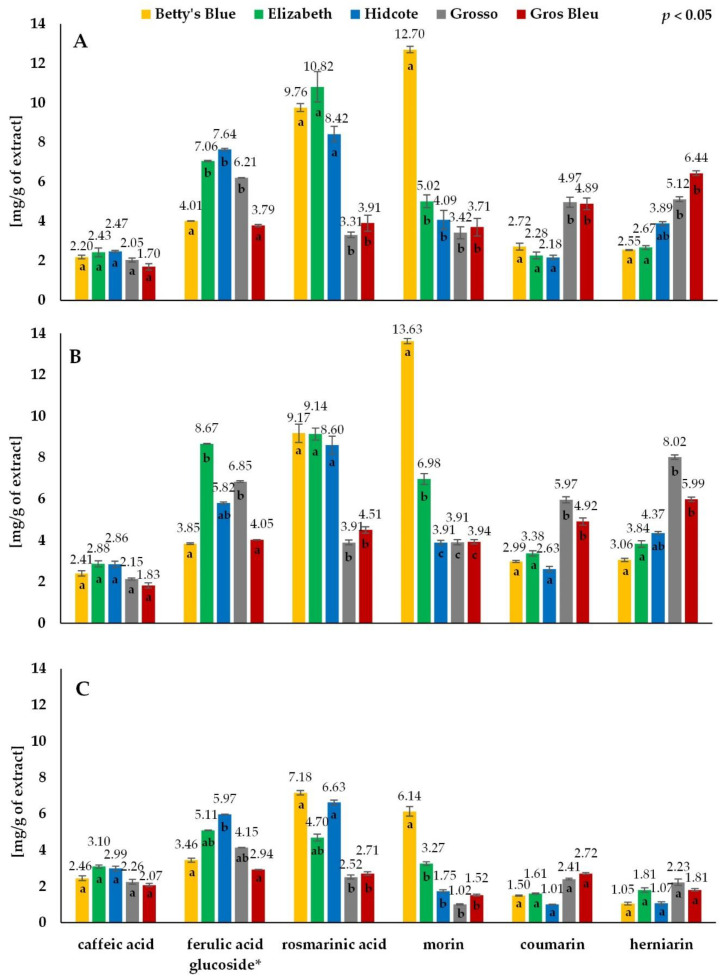
Quantitative comparison of phenolic compounds and coumarins found in aqueous-ethanolic (UAE (**A**) and maceration (**B**)) and aqueous (decoction) (**C**) extracts of lavender (Betty’s Blue, Elizabeth, Hidcote) and lavandin (Grosso, Gros Bleu). * The content of ferulic acid glucoside was expressed as ferulic acid equivalents. The values with different letters within the column designate statistically significant differences, *p* < 0.05 by ANOVA. Statistical analysis was performed separately for each compound.

All the chromatograms showed a similar profile of phenolic compounds and coumarins, as shown in Figure 4. However, differences were found in the content of individual phenolic compounds between the cultivars and the extraction method. The contents of phenolic compounds: caffeic acid (no. 2; CA), ferulic acid glucoside (no. 4; FA), rosmarinic acid (no. 9; RA), morin (no. 10; M) and coumarins: coumarin (no. 8; C) and herniarin (no. 11; H), as the most abundant ones, were analysed (Figure 5). The obtained results were calculated as mg/g of freeze-dried extract. The content of ferulic acid glucoside was expressed as ferulic acid. Amounts of rosmarinic acid (2.52–7.18 mg/g), ferulic acid glucoside (2.94–5.97 mg/g) and morin (1.02–6.14 mg/g) were lower in decoctions than in the ethanolic extracts: UAE (3.31–10.82 mg RA/g, 3.79–7.64 mg FA/g, 3.42–12.70 mg M/g) and macerates (3.91–9.17 mg RA/g, 3.85–8.67 mg FA/g, 3.91–13.63 mg M/g). On the other hand, there was no significant difference in the content of caffeic acid. The highest values for caffeic acid were obtained for decoctions (2.07–3.10 mg/g), followed by maceration (1.83–2.88 mg/g) and UAE (1.70–2.47 mg/g). In turn, significant differences were found between the extraction methods, especially between macerates (2.63–5.97 mg C/g and 3.06–8.02 mg H/g) and decoction (1.01–2.72 mg C/g and 1.05–2.23 mg H/g), for the content of coumarin and herniarin, respectively. Thus, it indicates that the best extraction method should be chosen considering the specific compounds and not the whole groups.

The content of rosmarinic acid, ferulic acid glucoside, morin and caffeic acid was higher in lavender (Betty’s Blue, Elizabeth, Hidcote), while the content of coumarin and herniarin was higher in lavandin (Grosso, Gros Bleu) in all types of extracts. The exception was the content of ferulic acid glucoside obtained for the Grosso cultivar, with a higher result for maceration (6.85 mg/g), UAE (6.21 mg/g) and decoction (4.15 mg/g) than for the Betty’s Blue cultivar, 3.85 mg/g, 4.01 mg/g and 3.46 mg/g, respectively (Figure 5).

The highest variance among cultivars of the same species was observed for morin, though only for lavender species, ferulic acid glucoside and herniarin. On the other hand, the rosmarinic acid and coumarin content differed between species but not between cultivars of the same species. Thus, it would indicate that the path of synthesis of these compounds is species-specific, not cultivar-specific.

### 3.3. Antioxidant Properties

**Figure 6:**
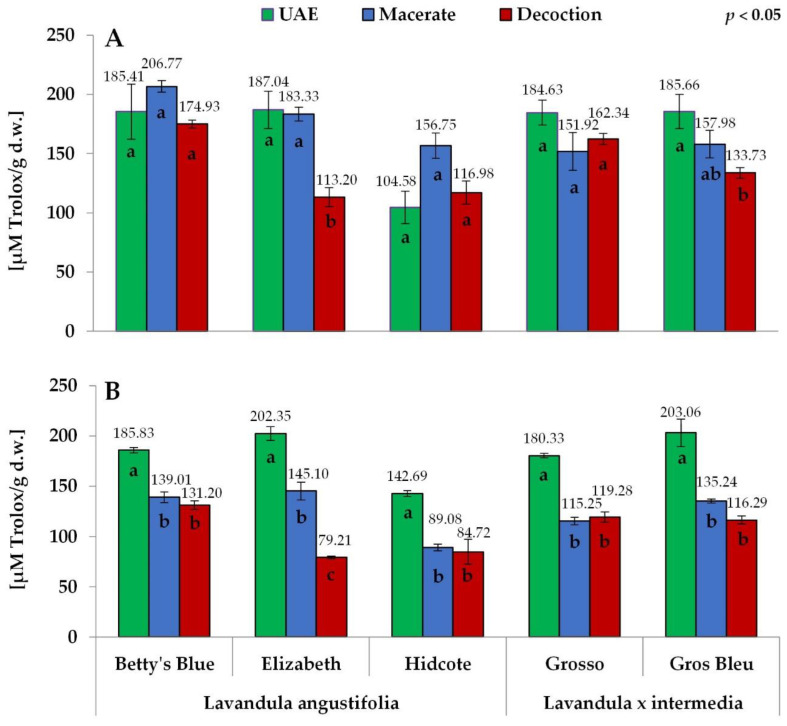
Antioxidant activity determined with DPPH (**A**) and FRAP (**B**) assays for different extraction methods. The values with different letters within the column designate statistically significant differences, *p* < 0.05 by ANOVA. Statistical analysis was performed separately for each cultivar.

The antioxidant properties were determined by two assays: DPPH for antiradical properties and FRAP for reducing properties. Both methods gave similar results (104.58–206.77 μmol Trolox/g d.w. and 79.21–203.06 μmol Trolox/g d.w. for DPPH and FRAP assay, respectively). The values obtained for macerates and decoctions by DPPH assay were significantly higher than those obtained by FRAP assay, whereas the extracts obtained in the ultrasound-assisted process (UAE) of both methods gave similar results (Figure 6).

Generally, the highest radical scavenging and reducing properties were observed for the extracts obtained in the ultrasound-assisted process (104.58–187.04 μmol Trolox/g d.w. and 142.69–203.06 μmol Trolox/g d.w. for DPPH and FRAP assay, respectively). The only exceptions were the values obtained in the DPPH assay for the Hidcote cultivar, with the highest result for macerate (156.75 μmol Trolox/g d.w. versus 104.58 μmol Trolox/g d.w. for UAE extract and 116.98 μmol Trolox/g d.w. for decoction). The differences were more prominent in FRAP assay results, where, for all cultivars, the reducing properties were significantly stronger for the extracts obtained in the ultrasound-assisted process than for both macerates and decoctions. The differences between FRAP results for the latter two extraction procedures were not significant in most cases, except for the Elizabeth cultivar (Figure 6).

Jiménez-Zamora et al. [11] reported a value of 3.43 mmol Trolox/L for lavender infusion in DPPH assay, which corresponds to 257.25 μmol Trolox/g d.w. for plant material. This value is higher than most values obtained in this work, which could be due to the use of a different extraction method. On the other hand, Ferreira et al. [42], in a DPPH assay, obtained results equal to 9.2 mg DPPH/g d.w. (which roughly corresponds to 10 μmol Trolox/g d.w.) for *Lavandula angustifolia* and to 28 mg DPPH/g d.w. (circa 30 μmol Trolox/g d.w.) for *Lavandula pedunculata* decoctions. The above values are much lower than those obtained in our study for decoctions, as well as for other extraction procedures.

The order of other cultivars depended on the extraction method. Among the extracts obtained by an ultrasound-assisted procedure (UAE), the extracts from Grosso, Betty’s Blue, Gros Bleu and Elizabeth cultivars gave almost identical results (DPPH assay: 184.63–187.04 μmol Trolox/g d.w., FRAP assay: 180.33–203.06 μmol Trolox/g d.w.), whereas the Hidcote cultivar extract showed lower results (DPPH assay: 104.58 μmol Trolox/g d.w., FRAP assay: 142.69 μmol Trolox/g d.w.) (Figure 6).

Among the macerates, the order depended on the assay used. Betty’s Blue, Elizabeth, Gros Bleu, Hidcote and Grosso was the order obtained by DPPH assay (from 206.77 μmol Trolox/g d.w., for Betty’s Blue to 151.92 μmol Trolox/g d.w. for Grosso), whereas in FRAP assay, the order was Elizabeth, Betty’s Blue, Gros Bleu, Grosso and Hidcote (from 145.10 μmol Trolox/g d.w., for Elizabeth to 89.08 μmol Trolox/g d.w. for Hidcote).

As for decoctions, the sample order observed for both assays was the same. The highest result was obtained for Betty’s Blue cultivar (174.93 and 131.20 μmol Trolox/g d.w. for DPPH and FRAP assay, respectively), followed by Grosso (162.34 and 119.28 μmol Trolox/g d.w.), Gros Bleu (133.73 and 116.29 μmol Trolox/g d.w.), Hidcote (116.98 and 84.72 μmol Trolox/g d.w.) and Elizabeth (113.20 and 79.21 μmol Trolox/g d.w.).

The variations in the order of studied cultivars depending on the assay could be due to different mechanisms of the assays. In the FRAP assay, there is only the electron transfer mechanism present, whereas, in the DPPH assay, there is usually a combination of hydrogen transfer and electron transfer mechanisms. That could also be due to a different reaction medium, namely the acidic aqueous solution in FRAP assay and methanol in DPPH assay, which could affect the activity of phenolic compounds present in the extracts.

### 3.4. Correlation between Antioxidant Activity and Phenolic Content

**Figure 7:**
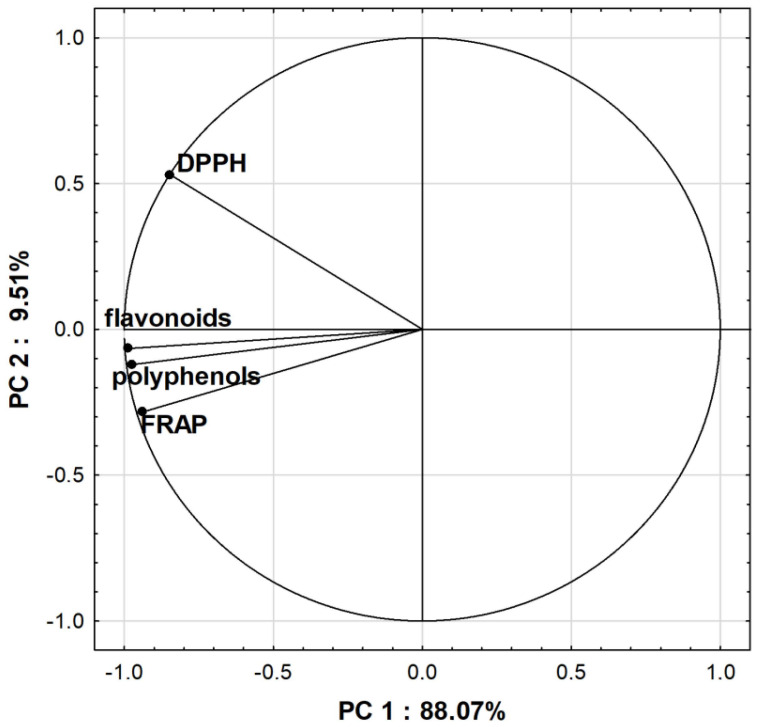
Correlation circle of the PCA for all extraction methods.

**Figure 8:**
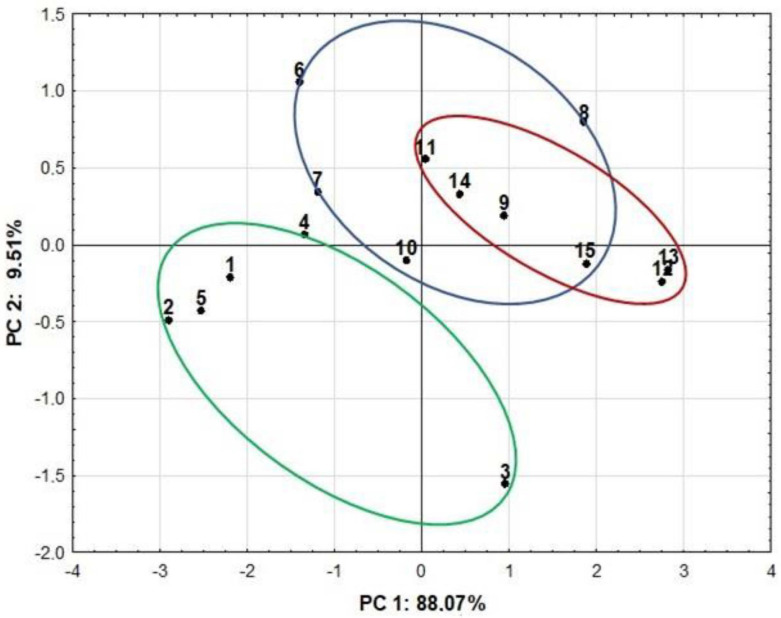
Score plot (PC 1/PC 2) of the PCA. Green circles, UAE; blue circles, macerates; red circles, decoctions. The marking of cultivars: 1,6,11—Betty’s Blue, 2,7,12—Elizabeth, 3,8,13—Hidcote, 4,9,14—Grosso, 5,10,15—Gros Bleu.

According to previous studies [11,12,13,43], antioxidant activity is associated with phenolic compounds. In our study, a highly positive correlation was observed between FRAP assay and total flavonoid contents. The highest Pearson’s correlation coefficient (r = 0.99) was obtained for the macerates, followed by the ultrasound-assisted method (UAE) (r = 0.97) and decoctions (r = 0.85). In addition, a significant positive correlation was found for the FRAP assay with the total phenolic content for macerates (r = 0.95) and the ultrasound-assisted method (r = 0.89). Our study also showed that the coefficient (r = 0.97) of correlation between the total phenolic content and the total flavonoid content for aqueous-ethanolic extracts was higher than for the aqueous extracts (r = 0.90). The correlation circle of the PCA (Figure 7) was also used to show correlations between the total content of polyphenols and flavonoids and antioxidant assays for all extraction methods. A positive correlation between flavonoids, polyphenols and FRAP was observed, while between flavonoids, polyphenols and DPPH, there was only a weak positive correlation. These variations could be related to the different mechanisms of action of antioxidant assays. The PCA was also a useful statistical tool to observe the separation between groups. The results are presented graphically as a score plot (Figure 8). PCA analysis showed that the first two principal components were able to explain 97.58% of the variability in the data. The first principal component (PC 1) described 88.07%, while the second (PC 2) was 9.51% of the total variance. The PCA allowed for confirming our previous results that the total content of polyphenolic compounds and antioxidant activity of extracts of lavender and lavandin cultivars depends dominantly on the cultivar and secondary on the extraction method. The most prominent effect was observed for ultrasound-assisted extracts, which were distinctly separated from macerates and decoctions.

## 4. Conclusions

The authors of this research investigated the effect of different extraction procedures on the recovery of the polyphenolic compounds of several cultivars of lavender (*Lavandula angustifolia* Mill.) and lavandin (*Lavandula* x *intermedia* Emeric ex Loisel.). HPLC analysis showed the presence of phenolic acids (rosmarinic acid, ferulic acid glucoside, caffeic acid, ellagic acid), flavonoids (morin, isoquercitrin, vanillin) and coumarins (herniarin, coumarin). In our study, the content of phenolic acids and flavonoids was higher in lavender (Betty’s Blue, Elizabeth, Hidcote), while the content of coumarins was higher in lavandin (Grosso, Gros Bleu) in all types of extracts. The extractability of bioactive compounds, both polyphenols and coumarins, depended on the extraction method as well. This effect was especially noticeable in the case of morin, rosmarinic acid, coumarin and herniarin. It could be due to such factors as the number of extraction steps, extraction time, solvent used and temperature. Too high temperature may contribute to the degradation of thermolabile compounds, which results in low extraction yields. In our study, aqueous-ethanolic extracts (UAE and macerates) showed a higher amount of bioactive compounds and antioxidant activity than aqueous extracts (decoctions). Our results indicated that the ultrasound-assisted extraction was the best method for extraction of polyphenolic compounds, while the macerate seems to be the optimum method for coumarin extraction. Furthermore, a good correlation was found between the total phenolic and total flavonoid contents and antioxidant activity (FRAP) of the lavender and lavandin extracts. To sum up, the results showed that the cultivar is the main factor differentiating the samples. Still, the extraction method significantly influences the composition and antioxidant properties of obtained extracts, even when the same solvent is used.

## Data Availability

Data is contained within the article and Supplementary Materials.

## Supplementary Materials

The following are available online at https://www.mdpi.com/article/10.3390/antiox11040711/s1. Figure S1: Chemical structures of the compounds of *Lavandula angustifolia* and *Lavandula* x *intermedia*: 1—chlorogenic acid, 2—caffeic acid, 3—vanillin, 4—ferulic acid glucoside, 5—ferulic acid, 6—ellagic acid, 7—isoquercitrin, 8—coumarin, 9—rosmarinic acid, 10—morin, 11—herniarin. Figure S2: UV spectra of phenolic compounds and coumarins. Figure S3: Mass spectrum of ferulic acid glucoside obtained for Betty’s Blue macerate. Table S1: Calibration curves, concentration range, limit of decection (LOD), and limit of quantification (LOQ) for standards. Table S2: Intra- and interday precision for determination of phenolic compounds by HPLC-DAD. Table S3: Compounds identified in aqueous-ethanolic and aqueous extracts of lavender and lavandin.
