# Supplementary material for: Phytochemical Profile and Antioxidant Activity of Lavandula angustifolia and Lavandula x intermedia Cultivars Extracted with Different Methods
Source: Antioxidants (Basel). 2022 Apr 5;11(4):711. doi: 10.3390/antiox11040711 (PMC9027103; doi:10.3390/antiox11040711)
Supplement: Supplementary file 1 [file antioxidants-11-00711-s001.zip › antioxidants-1649374-supp-back from 2nd proof.pdf]

Supplementary Materials

Table S1: Calibration curves, concentration range, limit of decection (LOD), and limit of quantification (LOQ) for standards.

Compound	Retention time (min.)	Calibration curve	R^2	Linear range (mg/mL)	LOD (mg/mL)	LOQ (mg/mL)
Caffeic acid	17.5	y = 49397035.98x + 4835.25	0.9987	0.01 - 0.10	0.008	0.024
Ferulic acid	31.1	y = 50589523.70x + 25423.55	0.9998	0.01 - 0.40	0.009	0.028
Coumarin	44.1	y = 12131245.04x + 83143.09	0.9986	0.01 - 0.20	0.016	0.047
Rosmarinic acid	45.9	y = 29357372.96x - 81691.18	0.9989	0.01 - 0.30	0.018	0.055
Morin	51.2	y = 15364684.92x - 65644.56	0.9988	0.01 - 0.30	0.020	0.059
Herniarin	55.0	y = 44968245.58x + 148823.98	0.9977	0.01 - 0.25	0.021	0.065

y – peak area (AU), x – concentration of the compound (mg/mL)

Table S2: Intra- and interday precision for determination of phenolic compounds by HPLC-DAD.

Compound	Concentration (mg/mL)	Intraday RSD (%)	Interday RSD (%)
Rosmarinic acid	0.040	0.288	0.326
	0.080	0.221	0.340
	0.200	0.182	0.317
Herniarin	0.040	0.064	0.198
	0.080	0.143	0.195
	0.200	0.045	0.057

Table S3: Compounds identified in aqueous-ethanolic and aqueous extracts of lavender and lavandin.

Peak number	Compound	Retention time (min.)	Molecular formula	Molecular weight (g/mol)	λ_{max} (nm) (A_{max})	Ion fragments
1	Chlorogenic acid	13.2	$C_{16}H_{18}O_9$	354.31	218 (1.15), 245 (0.81), 302 (1.10), 330 (1.44)	191, 179, 161
2	Caffeic acid	17.5	$C_9H_8O_4$	180.16	218 (2.11), 243 (1.59), 298 (2.13), 324 (2.61)	179, 134
3	Vanillin	24.2	$C_8H_8O_3$	152.15	206 (2.52), 230 (2.88), 278 (2.06), 308 (1.98)	151, 136, 123, 109
4	Ferulic acid glucoside	29.5	$C_{16}H_{20}O_9$	355.10	293 (-), 318 (-)	193, 149, 134
5	Ferulic acid	31.1	$C_{10}H_{10}O_4$	194.18	218 (1.76), 235 (1.59), 295 (1.83), 322 (2.40)	193, 178, 149, 134
6	Ellagic acid	31.9	$C_{14}H_6O_8$	302.19	202 (1.06), 252 (3.72), 366 (0.84)	301, 257, 229, 185
7	Isoquercitrin	35.3	$C_{21}H_{20}O_{12}$	464.40	207 (2.40), 257 (1.40), 358 (1.15)	300, 271, 255, 178
8	Coumarin	44.1	$C_9H_6O_2$	146.14	212 (2.93), 274 (2.26), 310 (1.16)	147, 119, 91
9	Rosmarinic acid	45.9	$C_{18}H_{16}O_8$	360.30	203 (1.26), 291 (0.46), 330 (0.62)	197, 179, 161,135
10	Morin	51.2	$C_{15}H_{10}O_7$	302.23	207 (2.31), 255 (1.17), 358 (0.91)	271,163
11	Herniarin	55.0	$C_{10}H_8O_3$	176.17	205 (2.48), 216 (2.22), 325 (2.58)	176, 148, 133

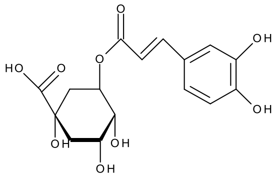

1

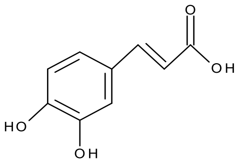

2

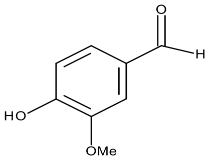

3

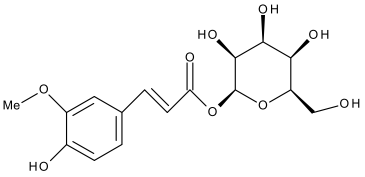

4

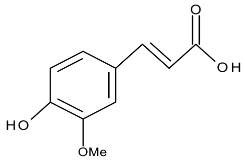

5

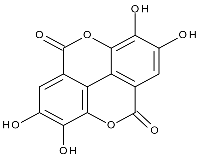

6

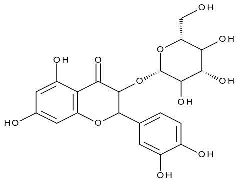

7

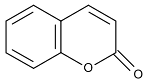

8

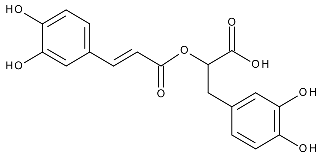

9

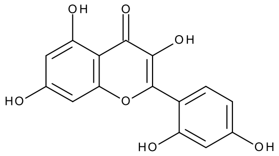

10

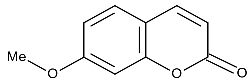

11

Figure S1: Chemical structures of the compounds of *Lavandula angustifolia* and *Lavandula* x *intermedia*: 1 - chlorogenic acid, 2 - caffeic acid, 3 – vanillin, 4 - ferulic acid glucoside, 5 - ferulic acid, 6 - ellagic acid, 7 – isoquercitrin, 8 – coumarin, 9 - rosmarinic acid, 10 – morin, 11 – herniarin

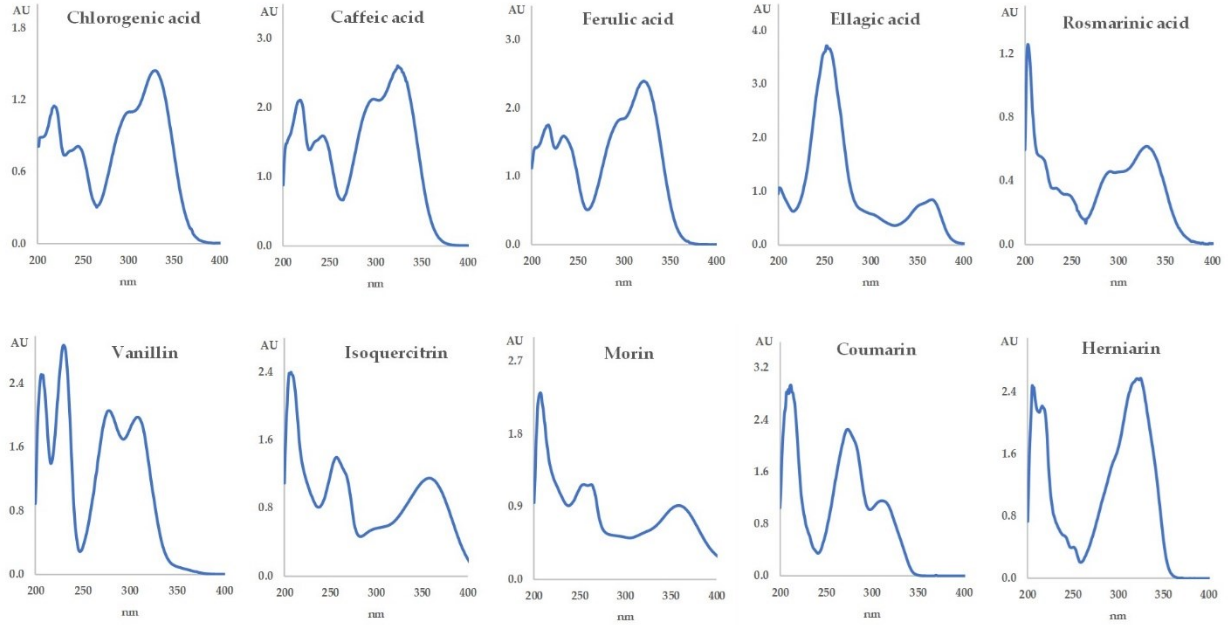

Figure S2: UV spectra of phenolic compounds and coumarins.

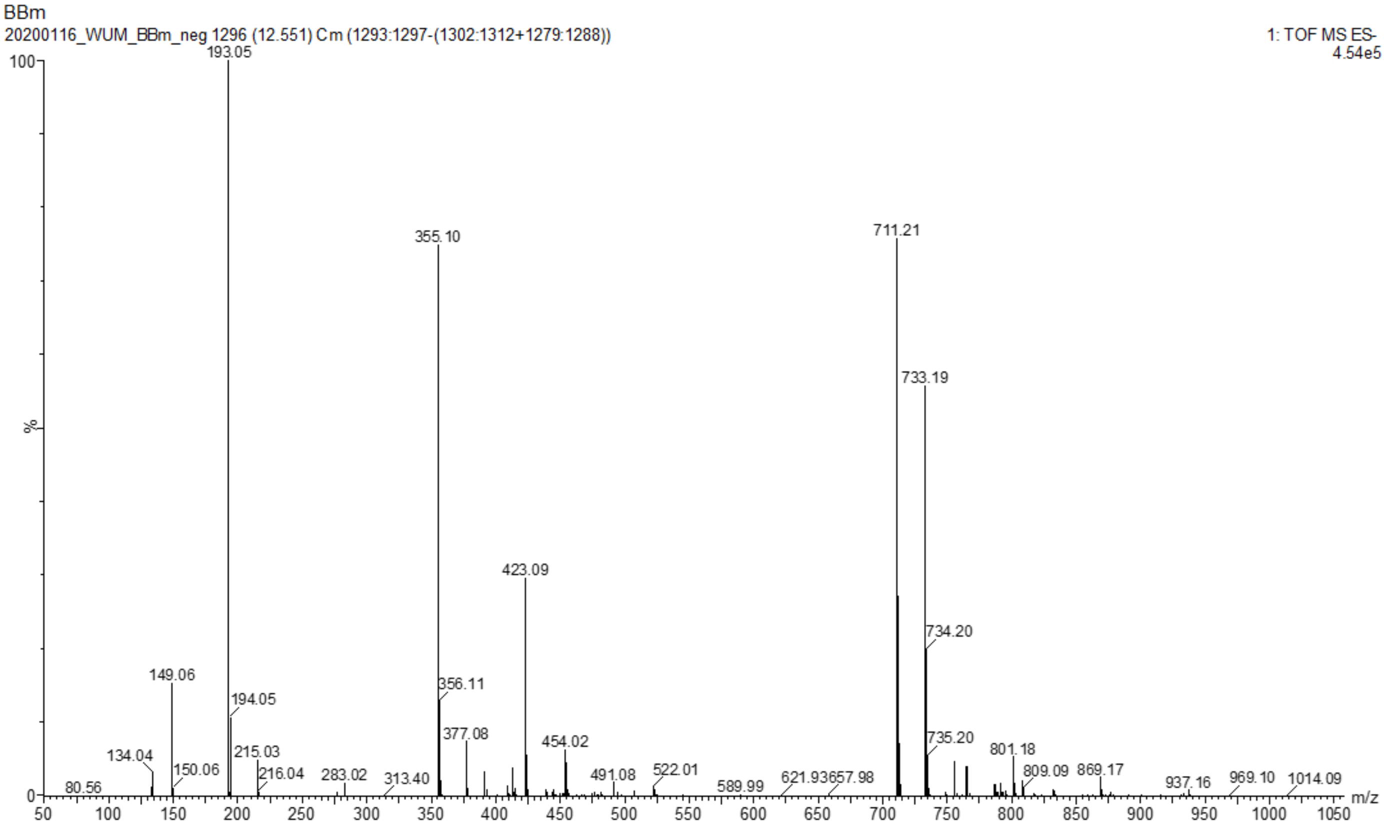

Figure S3: Mass spectrum of ferulic acid glucoside obtained for Betty’s Blue macerate.
